# Virtual group-based mindfulness program for autistic women: A feasibility study

**DOI:** 10.1177/17455057221142369

**Published:** 2022-12-22

**Authors:** Brianne Redquest, Ami Tint, Laura St. John, Sue Hutton, Pamela Palmer, Yona Lunsky

**Affiliations:** 1 School and Applied Child Psychology, Werklund School of Education, University of Calgary, Calgary, AB, Canada; 2 Alberta Children’s Hospital Research Institute, University of Calgary, Calgary, AB, Canada; 3 Azrieli Adult Neurodevelopmental Centre, Campbell Family Mental Health Research Institute, Centre for Addiction and Mental Health, Toronto, ON, Canada; 4 Department of Psychiatry, Temerty Faculty of Medicine, University of Toronto, Toronto, ON, Canada

**Keywords:** autistic women, feasibility, virtual mindfulness

## Abstract

**Background:**

Autistic women experience life differently than autistic men. For example, autistic women tend to be diagnosed significantly later than autistic men, they experience a higher number of traumas, and are at increased risk for mental health conditions. Given gender-specific life experiences, autistic women may benefit from gender-specific group-based supports. Virtual mindfulness has been shown to be helpful in improving well-being among autistic adults; however, limited research has explored the impact of virtual mindfulness when it is delivered to a group of autistic women only.

**Objectives:**

The aim of this article is to describe a preliminary evaluation of a virtual mindfulness group piloted for autistic women. Five key areas of feasibility were assessed in the current study: demand, implementation, acceptability, practicality, and limited efficacy testing.

**Methods:**

Twenty-eight women participated in a 6-week virtual autism-informed mindfulness program and were asked to complete measures assessing psychological distress, self-compassion, and mindfulness at pre and post. Participants were also asked to complete a satisfaction survey after the program.

**Results:**

Results showed that the program was feasible in terms of demand, implementation, practicality, and acceptability. While quantitative results showed there were no changes in psychological distress, self-compassion, and mindfulness from pre- to post-program, qualitative results showed some benefits.

**Conclusion:**

Given the unique challenges that some autistic women experience, offering groups to autistic women may have some value and it would be important to continue exploring this topic area.

## Introduction

Autism is a neurodevelopmental condition characterized by differences in social communication and restricted or repetitive behaviors.^1^ Autism has been referred to as a predominantly male condition, with a male to female ratio of 3:1.^2^ It is becoming increasingly recognized that some autistic girls and women present with subtle differences in autistic characteristics when compared with autistic boys and men.^3^ For example, autistic girls and women often have less overt restricted interests and greater awareness and desire for social interaction compared to autistic boys and men.^3–6^ As well, many autistic girls and women are better able to camouflage their autistic differences.^3^ Despite growing awareness of these sex/gender differences, autistic girls and women have been vastly underrepresented in both research and clinical practices, which has led to a male-centric understanding of autism. As a result, little is known about the experiences of autistic women or the services and supports that effectively meet their needs.

Emerging research highlights how the life course of autistic women often differs from autistic men. Autistic women tend to be diagnosed significantly later than autistic men, which is associated with challenges impacting identity, relationships, and mental health.^7^ Compared to autistic men, autistic women often encounter greater difficulty sustaining employment and experience more job instability.^8,9^ Similarly, autistic women experience a higher number of traumas and higher rates of sexual abuse than autistic men.^10^ In culmination of these risk factors, autistic women are also at increased risk for many mental health conditions with higher rates of psychiatric service use.^11,12^ Despite these significant needs, autistic women report more barriers to care than autistic men,^13^ with qualitative work highlighting their experiences of being the “autistic other” within male-dominated autism services.^14,15^

Recognizing the impact of gendered life experiences, there is growing support for gender-specific mental health service provision among the general population.^16,17^ Indeed, in mixed-gender treatment groups, some women report experiencing a lack of acceptance and understanding, inequitable “air time,” and negative experiences with men in the group, including unwanted sexual advances.^18,19^ In comparison, women-only groups have been cited to offer a unique treatment environment allowing for a greater focus on women’s issues and a more comfortable and safe setting in which women can discuss sensitive topics.^20^ The research to date has demonstrated positive outcomes and high attendance and retention rates for women-only groups in relation to substance use and gambling treatment.^21,22^ To our knowledge, no research to date has examined gender-specific group-based mental health treatment among autistic women, in either in person or virtual settings.

In 2018, our team of researchers, clinicians, and autistic advisors co-developed a virtual mindfulness program for autistic women and men based on a modified version of the Mindfulness-Based Stress Reduction (MBSR) curriculum.^23^ An evaluation of this program showed that participants reported reduced levels of distress which were maintained at 3-month follow-up, in addition to increased mindfulness, and self-compassion.^24^ While our initial evaluation did not include gender-based analyses, prior research exploring the impact of mindfulness on the mental health and quality of life among autistic adults suggests mindfulness may be a promising intervention for autistic women. Braden et al.^25^ showed that MBSR led to improvements in disability-related quality of life among autistic adults compared to a control intervention (support/education intervention). An exploratory analysis further revealed that only women in both intervention groups improved on physical health-related quality of life and women improved more than men on mental health-related quality of life.^25^

We delivered 10 mixed gender virtual mindfulness groups between September 2018 and January 2021, reaching 133 autistic adults from across Canada. Across these 10 groups, 6 groups had at least a 2:1 men to women ratio and 1 of the groups had only one woman of 16 participants. In running these groups, our team anecdotally observed how some autistic women appeared less engaged and interactive during discussions and activities within the larger group. There were also occasions where comments were made by men in the group, which resulted in women reporting discomfort to the group facilitators.

Acknowledging the growing literature base on gender-specific life experiences and needs of autistic women, and our team’s anecdotal reflections on the mixed-gender mindfulness program, we developed a virtual mindfulness program for autistic women only. The aim of this article is to describe a preliminary evaluation of this virtual mindfulness group.

## Methods

Using Bowen et al.’s^26^ framework for conceptualizing feasibility studies, five key areas were assessed in the current study. *Demand* was assessed in terms of number of participants recruited, and *implementation* was assessed by group attendance and dropout rate. *Acceptability* and *practicality* were assessed through open-ended questions from a satisfaction survey that asked what participants liked and did not like about the group. *Limited efficacy testing*, or whether the new program showed measurable change was considered in terms of changes in psychological distress (depression, anxiety, and stress), mindfulness, and self-compassion post-program, using a within subject’s design.

### Participants

Inclusion criteria to participate in the women’s mindfulness groups included being (1) 18 years or older, (2) proficient in English, (3) reside in Canada and (4) self-identified as an autistic woman. Participants were required access to a computer, tablet, or smartphone. People who did not identify as an autistic woman were not eligible to participate. We used the term “women” to refer to a broad gender categorization that includes all those who identify as women and is not restricted to sex assigned at birth. Further, participants who could not participate in the group independently were also not eligible to participate.

A total of 28 women registered to participate in one of two women’s mindfulness groups. All women who registered for the groups provided written informed consent to participate in research evaluating the program that was approved by the hospital Research Ethics Board. Participants ranged between 19 and 73 years of age, with a mean age of 35.9 years and came from four provinces across Canada (Ontario, Alberta, British Columbia, and New Brunswick).

### Procedure

#### Group description

The women’s only group was delivered following the same manualized approach as the mixed gender groups: autism-informed mindfulness for autistic adults. The group was delivered once a week for 60 min for 6 weeks. Main mindfulness practices included: eating meditation, breathing techniques (e.g. lotus breathing, figure eight), the body scan, mindful movement, and loving kindness. Participants were encouraged to participate in a way that they felt most comfortable. For some, this meant not turning on their camera, or using the chat box to share their thoughts, rather than unmuting themselves. The clinician facilitator (S.H.) received professional training from Jon Kabat-Zinn, the founder of mindfulness-based stress reduction. She has over 35 years of training in formal mindfulness practices, and 20+ years of experience in leading mindfulness groups to various communities, including those with disabilities and their families. A more detailed description of this program can be found in Lunsky et al.^24^

While the length of sessions, sequence, and topic selection were identical to the mixed-gender groups, there were a few key differences outside of content of the program. For example, in addition to ensuring that all participants identified as women, the clinician, autistic advisor, and technical support/course coordinator were women. Further, the mindfulness facilitator and the autistic advisor acknowledged that the group was for women only and emphasized during sessions that it was a safe space for women to share and to learn meditation techniques, and to receive support from other autistic women.

#### Recruitment

Participants were recruited through agencies across Canada that work with autistic people and their families between January and May 2021. It is worth noting that these groups were held during the second and third waves of COVID-19 in Canada. Interested participants contacted the researcher responsible for organizing the groups who then screened them for eligibility and obtained their written informed consent to participate. Survey measures were completed electronically one week prior to the first session and again in the week following the final session. Online surveys were completed through REDCap. Participants recieved a $30.00 honorarium for completing all research measures.

### Outcome measures

Participants were asked to complete the following measures prior-to and after the group:

*Depression Anxiety Stress Scales* (DASS-21).^27^ The DASS 21 is used to assess psychological distress. It includes three subscales each consisting of seven items; these subscales are a depression subscale, an anxiety subscale, and a stress subscale. Responses are recorded on a 4-point (0–3) Likert-type scale. A score of 0 indicated the statement “Did not apply to me at all” while 3 indicated that a statement “Applied to me very much” or “Most of the Time.” A total score was calculated by summing the items. Participant scores could range from a possible 0 to 64, with higher scores indicating higher levels of distress. Internal consistency of the DASS-21 was very good for the current sample at baseline (Cronbach’s α = 0.93).
*Patient Health Questionnaire- 9* (PHQ-9).^28^ The PHQ-9 is the 9-item depression scale of the patient health questionnaire. It is one of the most validated tools in mental health and can be a powerful tool to assist clinicians with diagnosing depression and monitoring treatment response. The nine items of the PHQ-9 are based directly on the nine diagnostic criteria for major depressive disorder in the DSM-IV. Total possible scores on the PHQ-9 could range from 0 to 27, with any score above 20 indicating severe depression. Internal consistency of the PHQ-9 was very good for the current sample at baseline (Cronbach’s α = 0.91).
*Generalized Anxiety Disorder Assessment* (GAD-7).^29^ The GAD-7 is a 7-item instrument that is used to measure or assess the severity of generalized anxiety disorder. Each item asks the individual to rate the severity of their symptoms over the past 2 weeks. Total possible scores on the GAD-7 could range from 0 to 21, with a score above 15 indicating severe anxiety. Internal consistency of the GAD-7 was very good for the current sample at baseline (Cronbach’s α = 0.96).
*Self-Compassion Scale- Short Form* (SCS-SF).^30^ The SCS-SF is a 12-item measure that uses a 5-point Likert-type scale (1 almost never to 5 almost always) to assess self-compassion. Items were totaled with reverse scoring being applied to three of the six subscales: self-judgment, isolation and overidentification. Positively scored subscales are common humanity, mindfulness, and self-kindness. Internal consistency of the SCS-SF was very good for the current sample at baseline (Cronbach’s α = 0.82).
*Five-Facet Mindfulness Questionnaire- Short Form* (FFMQ-SF).^31^ The FFMQ-SF is used to assess multiple aspects of mindfulness including non-reactivity (five items), observing (four items), acting aware (five items), describing (five items), and non-judgment (five items). The questionnaire consists of 24 items, each rated using a 5-point Likert-type scale (1 never to 5 always true). Internal consistency of the FFMQ-SF was very good for the current sample at baseline (Cronbach’s α = 0.83).

#### Program satisfaction

Participants were asked to complete a short survey regarding their satisfaction with the program. The survey consisted of 16 closed-ended questions pertaining to their experience within the program. These questions were about the content, virtual nature, and comfort within the group, as well as how connected they felt to the other participants. Also using closed-ended questions, participants were asked to rate their ability to engage with each practice. Using six open-ended questions, participants were asked to provide further feedback about what they liked and found helpful about the program, what they did not like and what they would change about the program, and if they had any concerns or comments about the program.

### Data analyses

*Demand* was assessed in terms of number of participants recruited, and *implementation* was assessed by group attendance and dropout rate. *Acceptability* and *practicality* were assessed by analyzing open-ended questions from the satisfaction survey. Due to brevity of responses, open-ended responses were summarized. For acceptability, quotes from participants are provided and pseudonyms are used.

Finally, *limited efficacy testing* was assessed using permutation analyses for paired data. A permutation analysis was chosen as it is appropriate for data which is unbalanced and not normally distributed. Changes in mean scores from pre-program to post-program scores were examined with a *p* value set to 0.05. Participants were excluded for the quantitative analysis if more than 30% of their data was missing (i.e. incomplete/non-respondent). Therefore, 16 women were included in these analyses. This sample size is appropriate given the aims of this pilot study.^32,33^ All analyses were conducted using R Studio software.

## Results

### Feasibility: demand and implementation

#### Demand

We delivered two virtual mindfulness groups for autistic women. The recruitment period for the first group was roughly 3 weeks, in which 13 participants were successfully recruited. Most of the women who registered for the second group came from a waitlist of participants (n = 9) who could not join the first group because they either could not attend during the time it was offered or because it was full (maximum enrollment was 15 participants). Six other people were recruited over approximately 3 weeks. Across these two groups, we were able to recruit 28 autistic women. Of these 28 women who consented to be in the project, 26 completed baseline measures and 16 completed post-measures. While 16 participants are appropriate given that the current study is a pilot study, a post hoc power analysis did indicate that the study was not adequately powered to detect meaningful change.

#### Implementation

**Figure 1.**
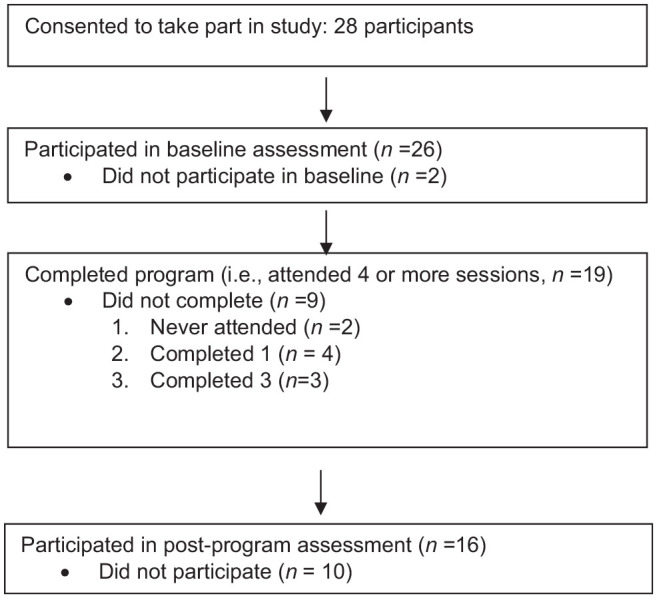
Recruitment.

Attendance at four or more of six sessions was considered completion. Nineteen of the 28 participants completed the program (four or more sessions); five attended three sessions; two attended one session, and two did not attend any sessions. Seven participants dropped out of the program: 2 of 13 from the first group, and 5 of 15 from second group. Reasons for dropout included being too busy and the program not being the right fit (see Figure 1).

### Feasibility: acceptability and practicality

Program feedback was collected through a satisfaction questionnaire that was completed by 12 of the 16 participants who completed post measures. Below is a summary of the feedback.

#### Practicality

##### Online nature of the groups

Eight participants agreed or strongly agreed that the online sessions were convenient and easier to attend than in-person sessions, as the online component removed potential barriers to participation such as transportation requirements. Only three participants agreed or strongly agreed that they would have preferred if the group was in-person. For additional information regarding the content of the group, please see Table 1.

**Table 1.** Closed-end questions pertaining to program experience (n = 12).

Variable^a^	M (SD)	Range
Program-specific items^a^		
When I heard about this research project, I was excited to participate.	4.25 (1.14)	1–5
It was easy to understand the content presented in my group.	4.25 (1.22)	1–5
The group content was relevant for me	4.17 (1.19)	1–5
I thought the group content was interesting.	4.42 (0.90)	2–5
I was provided with new information throughout the group.	4.33 (1.37)	1–5
The program addressed goals that were important to me.	4.42 (0.67)	3–5
The program gave me skills that I can use in my everyday life.	4.5 (0.91)	2–5
I plan to continue to use the skills I learned in the future.	4.25 (0.87)	3–5
I felt supported and valued throughout the program.	4.25 (1.22)	1–5
Technology-specific items^b^		
The web-based conferencing software was easy to use.	6.33 (1.16)	3–7
I did not encounter many technological issues while trying to participate in the sessions.	5.92 (1.24)	3–7
I was able to find a quiet place that allowed me to participate virtually in the sessions.	6.58 (0.67)	5–7
I felt connected to the other participants in this online group, and I thought there was a strong sense of community even though the sessions were virtual.	4.33 (1.83)	1–7
The online sessions were convenient and easier to attend than in-person sessions, as the online component removed potential barriers to participation such as transportation requirements.	5.33 (2.43)	1–7
My comfort level with the web-based technology has improved over the course of the sessions.	4.75 (1.87)	1–7
I would have preferred to be a part of an in-person group.	3.75 (2.18)	1–7

Higher ratings are indicative of more agreement with item.

a Items rated on a 5-point scale (1 = strongly disagree; 5 = strongly agree).

b Items rated on a 7-point scale (1 = strongly disagree; 7 = strongly agree).

##### Formal practices

As shown in Table 2, all of the practices were rated positively with mindful movement and figure 8 breathing having the highest ratings.

**Table 2.** Closed-end questions pertaining to practices (n = 12).

Ability to engage with practice^a^	M (SD)	Range
Figure 8 breathing	2.50 (.67)	1–3
Ocean breath	2.42 (.67)	1–3
Progressive muscle relaxation	2.33 (.78)	1–3
Body awareness	2.42 (.79)	1–3
Mindful movement (i.e. mindful walking)	2.50 (.80)	1–3
Loving kindness (i.e. metta)	2.23 (.75)	1–3

a 1 = could not engage with this practice at all, 2 = could engage with this practice to some degree, 3 = could easily engage with this practice.

#### Acceptability

##### What worked

Through open-ended questions, participants reported benefits associated with the group. Many participants reported that they appreciated learning new and relevant skills that they planned to use in the future. Elyse noted,

I plan on continuing to practice mindfulness as part of my daily routine and other aspects of my everyday life. I finally feel like I am able to find ways to accept myself, be more compassionate towards other people, and be more attentive to the present moment.

Participants noted the benefits of connecting with other autistic people, and in some cases, autistic women specifically. Cindy wrote, “They (autistic women) had gone through similar stuff that I have gone through and understand me better.” Participants further noted that they felt a sense of belonging and inclusion. Kim stated “[I] felt less alone and isolated with being autistic.” And finally, the importance of offering accessible supports was highlighted by Marjorie “It was important that it was at no cost, many folks cannot afford ‘help’, even on a sliding scale.”

##### What did not work

Through open-ended questions, participants reported challenges associated with the group, as well as recommendations for the future. A common challenge concerned the time of day in which the group was offered, either due to schedule conflicts or because of a lack of energy. Leah noted that a challenge for her was the chat: “I didn’t like the chat box during practice. I found it very distracting, and it seemed like a separate meditation practice to also not focus on chat conversation.”

In terms of what participants would change about the group and what would make it easier for them to participate, participants provided a range of answers; some of which included scheduling, for example having the sessions offered on a different day of the week. Other responses focused on the structure of the groups, as evident in the Lauren’s response: “I think it would be easier if the group stayed more on track and there was a bullet-point syllabus sent out the week or day before the group meeting.” It was also noted by participants that they felt the sessions were too short and that they would have liked the program to be longer than six weeks. In addition to making the sessions longer, participants suggested including practices to help to deal with loss and invasive thoughts.

### Reflections of group facilitators

Our team (S.H. and P.P.) anecdotally observed that, compared to prior groups which included men and women, autistic women appeared to be more interactive and comfortable in the autistic women’s only group. For example, women would comment on others’ shared experiences and in cases where women were emotional, the other women were quick to send a supportive message in the chat box. Some of the content of discussions in the autistic women’s groups differed from the discussions in mixed-gender groups. For example, several autistic women shared their experiences of obtaining their autism diagnosis and their emotions during that time, a topic that did not tend to come up in the mixed gender groups. In thinking about how the group might be adapted in the future, having more time for women to connect with one another was thought to be important.

### Feasibility: limited efficacy testing

Mean scores decreased from pre to post for the measures of psychological distress PHQ-9 (pre-mean = 9.3, post-mean = 8.2), GAD-7 (pre-mean = 9.8, post-mean = 7.9), and DASS-21 (pre-mean = 26.3, post-mean = 24.5), while they increased for the two mindfulness related scales SCS-SF (pre-mean = 36.2, post-mean = 37.2), and FFMQ-SF (total) (pre-mean = 75.4, post-mean = 77.8) measures. However, these changes were minimal and no statistically significant differences were found when comparing participants’ scores from pre-program to post-program (see Table 3).

**Table 3.** Pre- and post-scores across measures (n = 16).

	Pre	Post	*p*
PHQ mean score (SD)	9.3 (6.9)	8.2 (5.52)	0.47
GAD mean score (SD)	9.8 (7.8)	7.9 (7.0)	0.24
DASS mean score (SD)	26.3 (13.2)	24.5 (11.1)	0.32
SCS mean score (SD)	36.2 (9.7)	37.2 (8.8)	0.70
FFMQ mean score	75.4 (11.9)	77.8 (14.1)	0.29
Describe	15.13 (5.6)	14.2 (4.4)	0.46
Observe	14.8 (4.6)	15.9 (2.7)	0.35
Awareness	16.8 (3.6)	16.4 (3.5)	0.55
Non-reactivity	13 (4.6)	14.2 (2.8)	0.35
Non-judgment	15.6 (4.8)	17.1 (6.4)	0.31

PHQ: Patient Health Questionnaire; SD: standard deviation; GAD: Generalized Anxiety Disorder Assessment; DASS: Depression Anxiety Stress Scales; SCS: Self-Compassion Scale; FFMQ: Five-Facet Mindfulness Questionnaire.

## Discussion

This article provides a preliminary evaluation of a virtual mindfulness program piloted for autistic women. Results showed that the program was feasible in terms of demand, implementation, practicality, and acceptability; however, there were no changes in psychological distress, self-compassion, and mindfulness from pre- to post-program. Given the challenges that some autistic women have reported in accessing mental health supports,^13^ offering groups to autistic women may have some value and it would be important to continue exploring this topic area.

In contrast to our previous work examining the impact of mindfulness on a larger cohort of autistic adults,^24^ preliminary quantitative results from the current study did not reach statistical significance. While quantitative results showed no improvements, qualitative feedback suggested that the autistic women were satisfied and benefited from the group. Specifically, women described that the mindfulness practices were relevant and encouraged them to be present, compassionate toward others, and more accepting of themselves. Women further noted that they planned to continue to use mindfulness in the future. Prior to collecting further data, it would be worthwhile to work alongside an advisory of autistic women who can help to inform adaptations to the existing program. Both participants and facilitators thought, for example, that longer sessions might allow for more time to discuss related issues, such as the experience of being an autistic woman, or of getting a diagnosis.

Women in the current study appreciated connecting with other autistic women. Non-gender specific autism research has highlighted the benefits of peer support and connectedness within the autistic community.^34,35^ As well, gender-specific addiction treatment studies in non-autistic women have shown the value of women’s only treatments.^20,36,37^ It has been suggested that women’s only group therapy can feel safer, more supportive and more comfortable, and allow women to discuss gender-specific concerns. A randomized controlled trial comparing outcomes for women in mixed gender versus women’s only addiction treatment groups found that participants in the women only group made more supportive shared experience, and helpful strategy statements.^38^ How important being autistic, versus being the same gender, is for autistic women in group psychological treatments should be further explored. All of the women in the current study opted to register for this women’s only group but they were not asked whether they would be as likely to participate if the group was mixed gender. Assessing preferences of autistic women would be important in future work, as well as more explicitly measuring unique benefits of autistic women’s only therapeutic spaces, be they in person or virtual.

Findings from this study showed that most of the participants appreciated that the program was delivered online. This was also found in the Lunsky et al.^24^ study, in which participants described that the online nature of the program addressed barriers such as stresses associated with in-person communication and transportation. In addition, the online nature of the program allowed participants to meet autistic people from different provinces.^24^ As a result of the COVID-19 pandemic and related physical restrictions, several services for autistic people have transitioned from in-person to online. Benefits associated with online health services for autistic adults have been described in recent literature.^39,40^ Such benefits included reduced travel, improved comfort (avoiding crowded waiting rooms with sensory overload), and ease of communication. However, there are also several disadvantages associated with virtual delivery of supports and programs to autistic adults that should not be disregarded. Disadvantages include technology issues, environmental distractions, and reduced engagement.^24,39,40^ Further, virtual delivery excludes those who may require support with technology and do not have this support available to them, as well as those who do not have the access to a computer, tablet, phone, or Internet. It is important to recognize that online delivery of mindfulness as well as other interventions may benefit some autistic people, but not all. Future research could explore gender-specific preferences related to online individual and group treatments for autistic people.

### Limitations and future research

Limitations to this study should be taken into consideration when interpreting findings. First, participants self-identified as autistic and we did not differentiate between women with a formal diagnosis or a self-diagnosis. Future studies may wish to confirm diagnosis and look into differences between women who self-identify and those who have a formal diagnosis. While this may be considered a limitation, it is important to highlight that many autistic women have a difficult time getting diagnosed and can be misdiagnosed.^7^ The measures used in this study, although consistent with our prior research,^24^ were not autism specific. An important next step would be to utilize autism-specific measures of psychological distress, self-compassion, and mindfulness and to measure other relevant constructs such as connectedness within the autistic community. In the current study, there was a low completion rate on post-program measures and satisfaction surveys. Perhaps if measures were autism specific, and therefore more relevent to autistic women, completion rates would be higher.

While our study suggests that virtual mindfulness for autistic women is feasible, the clinical impact of an autistic women’s only virtual mindfulness group is not clear and requires further investigation. A first step is to work alongside autistic women to further adapt the virtual mindfulness program. Next, the impact of the adapted program could be assessed using a mixed methods approach that utilizes autism-specific measures and one-on-one semi-structured interviews. Future work could compare the experiences of women in an autistic-women only mindfulness group to women in a mixed-gender mindfulness group. A comparative study examining the impact of in-person versus virtual mindfulness groups for autistic women would also be valuable. Finally, it might be valuable to explore the impacts of other types of women’s only group treatments in the autism community, such as participating in group-based cognitive-based therapy and acceptance and commitment therapy.

## Conclusion

To our knowledge, this is the first study to examine the feasibility of a virtual mindfulness group for autistic women. The program was feasible in terms of demand, implementation, practicality, and acceptability. While quantitative results showed there were no changes in psychological distress, self-compassion, and mindfulness from pre- to post-program, qualitative results suggested some benefits. Important next steps include further adapting the program to meet the needs of autistic women and continued study. This must not overshadow the need to ensure that mental health supports for all autistic people are gender informed.

## Declarations

**Ethics approval and consent to participate:** All women provided written informed consent to participate in research evaluating the program that was approved by the Centre for Addiction and Mental Health Ethics Board (REB 116-2017).

**Consent for publication:** Informed consent for publication was provided by participants. Identifying details have been omitted.

**Availability of data and materials:** Participants of this study did not agree for their data to be shared publicly, so supporting data are not available.
